# Sexual function and sexual satisfaction in individuals undergoing infertility: A systematic review

**DOI:** 10.18502/ijrm.v21i9.14404

**Published:** 2023-10-30

**Authors:** Armin Firoozi, Farah Lotfi Kashani, Shahram Vaziri

**Affiliations:** ^1^Department of Psychology, Roudehen Branch, Islamic Azad University, Roudehen, Iran.; ^2^School of Medicine, Department of Psychology, Tehran Branch, Islamic Azad University of Medical Sciences, Tehran, Iran.

**Keywords:** Sexual health, Sexual satisfaction, Psychological intervention, Early intervention, Educational.

## Abstract

**Background:**

Although sexual function (SF) and sexual satisfaction (SS) are the essential factors influenced negatively by infertility and may be associated with delaying or disrupting infertility treatment, no systematic review has assessed the results of these studies so far.

**Objective:**

The present study aimed to systematically review published interventional research regarding SF and SS among infertile individuals.

**Materials and Methods:**

In this systematic review, the databases of PubMed, MEDLINE, Web of Science, Scopus, ScienceDirect, Cochrane Library, PsycInfo, Google Scholar, and SID were searched to retrieve the relevant studies in Persian and English languages up to August 2021.

**Results:**

23 interventional studies were included in this systematic review, of which only 2 were low quality based on the critical appraisal skills program checklist. The interventions of the included studies were classified into 2 main categories: educational and psychological interventions for increasing the SF and SS among infertile women or couples.

**Conclusion:**

There was no clear evidence to understand the most effective method for increasing SF and satisfaction among couples with infertility. Based on the results of the included studies, all of them showed the effectiveness of the various interventions performed on sexual health dimensions among couples with infertility. This systematic review showed that most SF studies were educational, whereas the SS were more psychologically interventional. Conducting standardized and high-quality randomized controlled trials focusing on SF and SS is recommended.

## 1. Introduction

The World Health Organization and the American Society for Reproductive Medicine defined infertility as a condition where couples cannot achieve pregnancy despite 12 months of regular unprotected sexual intercourse (1, 2). The prevalence of infertility in developed countries ranges from 3.5-17%, estimated to be 6.9-9.3% in developing countries (3, 4). Also, in the Middle East, the prevalence of infertility has been estimated at 10-15% (5). Overall, infertility as a common public health concern can lead to psychological, socioeconomic problems, and sexual disorders among couples with infertility (2, 3). Studies showed that due to couples with infertility being exposed to various difficulties in adjustment to their situation, such as unsuccessful treatment and continuous lack of conception and childbearing, their sexual relationships, such as sexual function (SF) and sexual satisfaction (SS), may be negatively influenced (2, 6).

SS is an essential factor in a couple's health. It defined how the sexual partners' expectations can be met and how their reduction may be associated with adverse effects on the couple's body and mind (7, 8). The association between infertility and SS has been empirically addressed in the previously published literature. The studies showed that infertility can adversely affect SS among couples involved in the infertility treatment (6, 9). The literature review revealed that approximately 50-60% of couples with infertility reported a considerable reduction in their SS (10).

SF and SS are considered essential factors commonly influenced negatively by infertility among couples and may be associated with delaying or disrupting infertility treatment, so investigating these variables and paying attention to them can be effective. Although various studies with different research designs had been conducted regarding the SF and SS and their associated factors among couples with infertility, no systematic review assessed these variables among couples with infertility. Thus, the present study aimed to systematically review the published interventional studies on SF and SS among infertile individuals.

## 2. Materials and Methods

### Design

This systematic review was performed in August 2021 based on the preferred reporting items for systematic reviews and meta-analyses (PRISMA) guidelines (11, 12) for the integrative review of the published interventional studies regarding SF and SS among infertile individuals.

### Data source and search strategy

A literature search was conducted in electronic databases, including PubMed, MEDLINE, Web of Science, Scopus, ScienceDirect, PsycInfo, Cochrane Library, Google Scholar, and SID to write this systematic review. The latest search was performed separately between July and September 2021 by 2 researchers. The search process was followed according to the systematic review searches guidelines using Persian and English keywords as follows: [“clinical trial" OR “randomized controlled trial" OR “quasi-experimental" OR “non-randomized trial" OR “interventional studies"] AND [“sexual function" OR “sexual dysfunction" OR “sexual disorder" OR “sexual activity" OR “sexual problems" OR “SS" OR “sexual health satisfaction" OR “marital satisfaction" OR “couple's sexual satisfaction"] AND [“couples with infertility" OR “infertile women" OR “infertility in women" OR “infertile men" OR “infertility in men" OR “infertility" OR “infertility period" OR “primary infertility" OR “secondary infertility"] AND [“psychological intervention" OR “behavioral cognitive therapy" OR “supportive therapy" OR “integrative behavioral couple therapy" OR “couple therapy" OR “emotionally focused couples therapy" OR “infertility counseling" OR “sexual counseling" OR “health-education program" OR “educational intervention" OR “marital relationship enrichment program" OR “acceptance and commitment therapy" OR “reality therapy" OR “communication skills training" OR “sexual skills training" OR “sexual education" OR “psychosexual therapy" OR “lifestyle intervention"].

### Study selection

The references of the included studies were also searched to find more relevant articles. The titles and abstracts of the selected studies were independently screened by 2 researchers (AF and FLK). The full text of the studies with relevant abstracts was extracted and evaluated for further assessment based on the inclusion and exclusion criteria.

### Inclusion and exclusion criteria

Inclusion criteria included trials published in valid journals, such as single- or double-blind clinical studies, randomized controlled trials (RCTs), quasi-experimental research, pilot studies, etc., that presented original findings; performed non-pharmacological interventions on couples with infertility, men and/or women, adopted validated measures in the assessment of SF and SS, were published in Persian and English without any time limitations, and were conducted on couples with primary or secondary infertility. Studies used pharmacological agents for improving SF in couples with infertility or performed on couples with psychiatric disorders or psychological problems, and studies with other research designs such as qualitative, case reports, systematic review, editorial, cross-sectional and cohort studies were excluded from this systematic review.

### Data extraction and analysis

The full texts of the included studies were studied, and relevant information was extracted and provided in the organized tables and then checked by FLK. In the methodology process, each disagreement was resolved via discussions, and in the required situation, advice and comments of the third author were used. Information extracted included the author's name, country, publication year, type of trial, the sample size in each group (i.e., intervention and control groups (CG)), primary outcomes, mean ± standard deviation (SD) of SF and SS before and after the intervention, type, and duration of intervention, outcome measurement, and time of outcome measurement and results (Table I, II).

**Table 1:** Characteristics of the included studies regarding interventional studies on the SF of infertile individuals

**Author,** **country, year** **(Ref)**	**Type of trial**	**Sample size in each group**	**Primary outcome**	**Type of intervention**	**Duration of intervention**	**Participant's age groups or mean ± SD (yr)**	**The mean ± SD of SF in each group before the intervention**	**The mean ± SD of SF in each group after intervention**	**Outcome measurement**	**Summary of results**
**Fahami ** ***et al.*** **,** **Iran, 2015 (13) **	Clinical pretest-post-test field trial	IG: 32 CG: 32	SF in infertile women	CST workshop psycho-educational sessions	5, 3 hr weekly sessions	IG: 33.40 ± 7.60 CG: 32.50 ± 6.40	IG: 8.26 ± 9.40 CG: 8.26 ± 1.50	IG: 9.29 ± 1.40 CG: 6.26 ± 9.40	FSFI	CST increased the SF in infertile women in the IG compared to the CG (p < 0.001)
**Jalilian ** ***et al.*** **,** **Iran, 2018 (14)**	Quasi-experimental	IG: 20 CG: 20	Sexual dysfunction among infertile women	SST	10 weekly 2-hr sessions	22-36	IG: 28.70 ± 6.65 CG: 32.10 ± 7.27	IG: 70.00 ± 9.84 CG: 26.20 ± 10.53	FSFI	SST significantly affects sexual dysfunction scores in the IG compared to CG (p < 0.001)
**Jamali ** ***et al.*** **,** **Iran, 2018 (15) **	3-group RCT	IG1: 37 IG2: 37 CG: 37	The anxiety and regulating SF in infertile women	Personal training and educational booklet distribution strategies	Personal training group, 8 sessions (2 times per week over 4 wk) for 45 min	WIG1: 26.43 ± 3.89 MIG1: 30.89 ± 4.16 WIG2: 26.94 ± 5.52 MIG2: 31.94 ± 6.43 WCG: 27.91 ± 4.43 MCG: 31.91 ± 3.36	IG1: 14.80 ± 3.16 IG2: 15.73 ± 4.19 CG: 15.24 ± 2.56	IG1: 19.70 ± 2.17 IG2: 20.17 ± 2.37 CG: 14.85 ± 2.55	FSFI	There were significant differences between IGs and CG in 5 domains of SF: desire, arousal, lubrication, orgasm, and dyspareunia
**Ramadan ** *et al.*, **Egypt, ** **2018 (16)**	Quasi-experimental pre and post-intervention	IG: 100 CG: -	Sexual skills for improving SF	Educational intervention	4 scheduled sessions 0.5-1 hr	IG: 27.89 ± 8.06 CG: -	- -	FSFI	The educational intervention was statistically significant (p < 0.01) on infertile women's SF scores pre and post-one months of intervention
**Hasanzadeh** ***et al.*** **, Iran, ** **2018 (17)**	Quasi-experimental pretest-posttest	IG: 15 CG: 15	SF in infertile women	TA therapy	8 sessions, 2 hr weekly	20-40	IG: 56.93 ± 9.75 CG: 44.93 ± 13.07	IG: 60.46 ± 5.50 CG: 49.00 ± 12.23	FSFI	TA therapy in the posttest stage significantly affects SF (p < 0.05)
**Wekker ** ***et al.*** **,** **Netherlands,** **2018 (18)**	Multicenter RCT	IG: 272 CG: 278	Sexual the function of women with obesity and infertility	Lifestyle intervention	6 face-to-face consultations of approximately 30 min for 4 wk	IG: 30.20 ± 4.10 CG: 29.70 ± 4.30	- IG: 96.50 ± 14.20 CG: 91.40 ± 12.80	MFSQ	Total SF scores were significantly increased in IG compared to CG (p = 0.04)
SF: Sexual function, SD: Standard deviation, IG: Intervention group, CG: Control group, CST: Communication skills training, FSFI: Female sexual function index, SST: Sexual skills training, RCT: Randomized controlled or clinical trial, WIG: Women in the intervention group, MIG: Men in the intervention group, WCG: Women in the control group, MCG: Men in the control group, TA: Transactional analysis, MFSQ: McCoy female sexuality questionnaire										

**Table 2:** Characteristics of the included studies regarding interventional studies on SS of infertile individuals

**Author,** **country, year** **(Ref)**	**Type of trial**	**Sample size in each group**	**Primary outcome**	**Type of intervention**	**Duration of intervention**	**Participants' age groups or mean ± SD (yr)**	**The mean ± SD of SS in each group before the intervention**	**The mean ± SD of SS in each group after the intervention**	**Outcome measurement**	**Summary of results**
**Pakgohar** ***et al.*** **, Iran,** **2008 (19)**	RCT	IG: 50 CG: 50	SS among infertile women	Counseling	4 sessions, 1 hr once a week	IG: 26.88 ± 4.23 CG: 27.44 ± 4.65	- -	SSQ	Counselling significantly increased the SS in the IG compared to CG 3 months after the intervention (p = 0.01)
**Hussein Iraq,** **2014 (20)**	RCT	IG: 70 CG: 70	The marital satisfaction rate of couples with infertility	Psychological intervention	6-month psychological treatment with CBT, supportive psychotherapy	WIG: 27.00 ± 4.20 MIG: 31.00 ± 4.24 WCG: 26.00 ± 5.19 MCG: 31.00 ± 6.22	IG: 146.56 ± 18.06 CG: 154.92 ± 19.59	IG: 166.69 ± 20.63 CG: 147.52 ± 21.38	ENRICH marital satisfaction questionnaire	A significant decrease in marital satisfaction over time in the CG and a considerable increase in the study group (p < 0.001)
**Soleimani** ***et al.*** **, Iran,** **2015 (21)**	Quasi-experimental	IG: 30 CG: 30	SS and marital adjustment of couples with infertility	EFT-C	10 sessions 120 min	33.8 ± 5.03	IG: 129.4 ± 19.87 CG: 121.47 ± 16.73	IG: 163.12 ± 22.51 CG: 119.21 ± 14.37	Index of SS	Results showed that EFT-C significantly impacted SS in IG compared to CG (p = 0.02)
**Solati ** ***et al.*** **,** **Iran, 2016 (22)**	Quasi-experimental	IG: 20 CG: 20	Marital satisfaction in infertile women	Stress management based on group CBT	10 two-hr	IG: 29.4 ± 0.00 CG: 28.01 ± 0.00	IG: 28.20 ± 7.20 CG: 30.06 ± 7.38	IG: 28.20 ± 7.20 CG: 30.06 ± 7.38	ENRICH marital satisfaction inventory	Marital satisfaction scores were significantly different between the IG and CG in both post-test (p < 0.001) and follow-up stages (p < 0.001)
**Masoumi ** ***et al.*** **,** **Iran, 2017 (23)**	RCT	IG: 50 CG: 50	Marital satisfaction, marital intimacy, and SS	MREP	Seven 90 min sessions	IG: 30.00 ± 4.90 CG: 28.30 ± 4.40	IG: 57.80 ± 18.00 CG: 26.40 ± 11.00	IG: 84.00 ± 1.20 CG: 39.30 ± 18.00	Linda Berg's SS	The pretest and post-test mean scores showed that the MREP significantly increased the SS among IG compared to CG (p < 0.001)
**Latifnejad** **Roudsari** ***et al.*** **,** **Iran, 2017 (24)**	RCT	IG: 29 CG: 31	Marital satisfaction in infertile women	Collaborative infertility counseling	45-60 min 8-9 wk	20-40	IG: 24.78 ± 13.20 CG: 23.74 ± 11.59	IG: 22.86 ± 15.01 CG: 26.61 ± 14.20	MSI	The IG's baseline mean marital satisfaction score after the intervention showed the mean difference between the groups was significant (p = 0.02)
**Ashrafian** ***et al.*** **, Iran,** **2019 (25)**	Quasi-experimental	IG: 15 CG: 15	SS and marital adjustment of an infertile woman	Integrative positive-CBT	10 sessions 90 min	25-40	IG: 44.74 ± 5.52 CG: 42.67 ± 5.45	IG: 66.07 ± 4.79 CG: 44.60 ± 5.99	SSQ	Teaching an integrated approach to positive CBT increased SS of infertile women in the experimental group compared to the CG (p < 0.01)
**Shahbazi ** ***et al.*** **,** **Iran, 2020 (26)**	Quasi-experimental	IG: 40 CG: 40	Satisfaction with quality of sexual relationship among women with infertility	Health-education program based on the BASNEF model	4 sessions of 60-90 min	IG: 31.90 ± 5.54 CG: 31.40 ± 6.00	- -	Researcher made questionnaire	After the intervention, satisfaction with the quality of sexual relationships had significantly improved in the experimental group compared to CG (p < 0.05)
**Kim ** ***et al.*** **,** **South Korea,** **2020 (27)**	Quasi-experimental	IG: 26 CG: 24	Infertility, stress, depression, intimacy, SS, and fatigue	Psychological intervention	6 sessions, each session 4 hr weekly	< 34- ≥ 35	IG: 2.79 ± 0.61 CG: 2.88 ± 0.51	IG: 3.66 ± 0.39 CG: 3.08 ± 0.28	DSFI	The experimental group demonstrated significant improvements in SS (z = 3.148, p < 0.001) compared to CG
**Vazirnia ** ***et al.*** **,** **Iran, 2021 (28)**	Single-case experimental study	IG: 6 CG: -	Infertility self-efficacy, dyadic adjustment, and SS	IBCT	Twelve 120-min sessions	34.00 ± 8.60	- -	SSQ	The provided IBCT increased SS (55.01%) in couples with infertility
SS: Sexual satisfaction, SD: Standard deviation, RCT: Randomized controlled or clinical trial, IG: Intervention group, CG: Control group, SSQ: Larson SS questionnaire, CBT: Cognitive-behavioural therapy, WIG: Women in the intervention group, MIG: Men in the intervention group, WCG: Women in the control group, MCG: Men in the control group, ENRICH: Evaluation and nurturing relationship issues, communication, and happiness, EFT-C: Emotionally focused couples therapy, MERP: Marital relationship enrichment program, MSI: Marital satisfaction index, DSFI: Dergatis sexual function inventory, BASNEF: Beliefs, attitudes, subjective norms, and enabling factors, IBCT: Integrative behavioral couple therapy										

### Type of outcome measure 

The primary outcome measured in this study was systematically assessing the interventions regarding SF among infertile women or couples. The secondary outcome was systematically investigating the interventions regarding SS among infertile women or couples.

### Quality assessment of (risk of bias) the included studies

To assess the quality of the studies included in this systematic review, the critical appraisal skills programme (CASP) checklist for RCTs was systematically utilized. The CASP system is well known to many healthcare practitioners and is widely used to critique studies and assessments in health and social care (29). This tool comprises 9 questions, and the authors (AF and FLK) independently evaluated the included studies and graded them using the CASP quality assessment criteria for RCTs. According to the strengths and weaknesses of the studies, the CASP was graded as “High", “Moderate", and “Low". The tool generates binary scores: 1 for “satisfied" and 0 for “unsatisfied" items (30) (Table III).

**Table 3:** Quality assessment of included studies by the critical appraisal skills program


**First author (yr) (Ref)**	**The study focused issue**	**Randomized assignment of patients**	**Did the proper selection of patients**	**Blinded experiment**	**Identified similarity of the groups at the beginning of the trial**	**Treated the groups equally**	**Applied results in the context**	**Considered clinically important outcomes**	**Weighted for benefits over harms and costs**	**Quality**
**Pakgohar (2008) (19)**	1	1	1	0	1	1	1	1	1	High
**Hussein (2014) (20)**	1	1	1	0	1	1	1	1	1	High
**Fahami (2015) (13)**	1	1	1	0	1	1	1	1	1	High
**Soleimani (2015) (21)**	1	1	1	0	1	1	1	1	1	High
**Solati (2016) (22)**	1	1	1	0	1	1	1	1	1	High
**Masoumi (2017) (23)**	1	1	1	1	1	1	1	1	1	High
**Latifnejad Roudsari** **(2017) (24)**	1	1	1	0	1	1	1	1	1	High
**Jalilian (2018)** **(14)**	1	1	1	0	1	1	1	1	1	High
**Jamali (2018) (15)**	1	1	1	0	1	1	1	1	1	High
**Ramadan (2018) (16)**	1	0	0	0	1	0	1	1	1	Low
**Hasanzadeh (2018)** **(17)**	1	1	1	0	1	1	1	1	1	High
**Wekker (2018) (18)**	1	1	1	0	1	1	1	1	1	High
**Karakas (2019) (31)**	1	0	1	0	1	1	1	1	1	Moderate
**Hasanzadeh (2019)** **(32)**	1	1	1	0	1	1	1	1	1	High
**Sahraeian (2019) (33)**	1	1	1	0	1	1	1	1	1	High
**Marvi (2019) (34)**	1	1	1	1	1	1	1	1	1	High
**Ashrafian (2019) (25)**	1	1	1	0	1	1	1	1	1	High
**Pasha (2020) (35)**	1	1	1	0	1	1	1	1	1	High
**Shahbazi (2020) (26)**	1	0	1	0	1	1	1	1	1	Moderate
**Kim (2020) (27)**	1	0	1	0	1	1	1	1	1	Moderate
**Mohammadzadeh** **(2021) (36)**	1	1	1	0	1	1	1	1	1	High
**Alimanesh (2021)** **(37)**	1	1	1	0	1	1	1	1	1	High
**Vazirnia (2021) (28)**	1	0	0	0	0	0	1	1	1	Low

### Ethical considerations

The research was conducted following the ethical principles and the national standards for conducting medical research.

## 3. Results

### Search results

**Figure 1:**
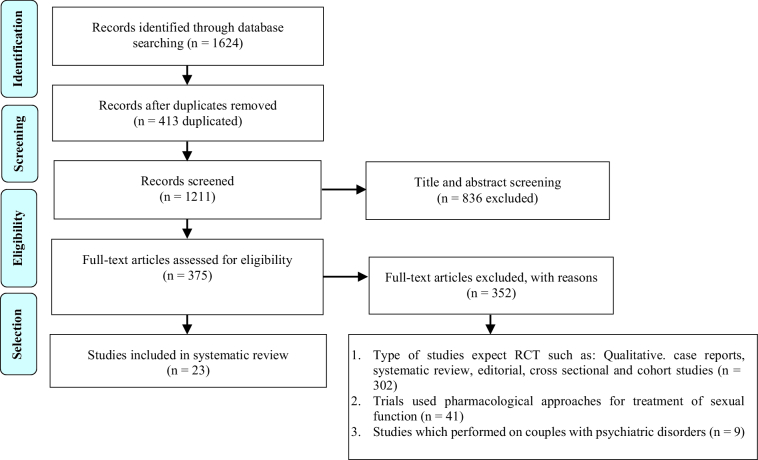
A flow chart of study selection according to PRISMA guidelines is presented.

The search yielded 1624 original research studies, of which 413 articles were removed due to duplicate results. The remaining 1211 articles were screened using their titles and abstracts, and 836 were excluded. In the final assessment step, the full text of the remaining studies was screened carefully according to the inclusion and exclusion criteria, and studies with cross-sectional, cohort, case-control, or review design (n = 302) used the pharmacological approach for the treatment of sexual dysfunction (n = 41), and also studies which were performed on couples with psychiatric disorders (n = 9) were excluded from this systematic review. Finally, 23 articles were selected and systematically reviewed. The PRISMA flowchart shows the selection process (Figure 1).

### Description of the included studies characteristics 

The results of the included articles are shown in table I. Of the 23 selected articles, 18 studies were conducted in Iran (13-15, 17, 19, 21-26, 28, 32-37), one in Korea (27), one in Iraq (20), one in Turkey (31), one in Egypt (16), and one in the Netherlands (18). The included articles were published between 2008 and 2021. The 23 studies included 2073 participants, and the sample sizes varied from 6-278 individuals. Of the 23 studies, 11 studies were randomized clinical or controlled trials (15, 18-20, 23, 24, 33-37), one clinical field trial (13), 9 quasi-experimental (14, 16, 17, 21, 22, 25-27, 32) and 2 experimental studies (28, 31). Of 23 included studies, 13 assessed the SF of couples with infertility or infertile women (13-18, 32-38), and 10 investigated the SS of couples with infertility or infertile women (19-28). From 13 studies investigating the SF as the main outcome measure, 12 studies used the female SF index (FSFI) to measure the SF of infertile women or couples with infertility before, and after the intervention (13-17, 31-37) and only in one study, McCoy female sexuality questionnaire (MFSQ) was used (18). Also from 10 studies that investigated the SS among couples with infertility or infertile women, 3 studies used Larson SS questionnaire (SSQ) for outcome measurement (19, 25, 28), 2 studies assess the SS through ENRICH marital satisfaction inventory (20, 22) and other studies used different measurements to measure the SS. Only in one study did the researcher made a questionnaire designed to investigate the SS that its validity and reliability were determined (26).

### The interventions regarding SF among infertile individuals 

The interventions of the included studies were classified into 2 main categories were reported below:

#### Educational interventions 

A total of 8 included studies investigated the use of different educational interventions to improve SF among couples with infertility or infertile women.

In the study investigating the effect of a communication skills training (CST) program on SF of infertile women, 32 couples with infertility were randomized into the control and training groups. The intervention group (IG) received education for 5 (3 hr) sessions weekly. The results showed that before the intervention, the mean scores of women's SF in both intervention and CGs had no significant differences (p = 0.997). At the same time, CST increased the SF in infertile women so that after training, the women's SF was significantly higher in the IG compared to the CG (p = 0.002) (13).

In the study, the effectiveness of sexual skills training (SST) using a cognitive-behavioral approach on sexual dysfunction of infertile women was assessed. This study randomly assigned 40 infertile women, for experimental and CGs at the infertility center. The patients in the IG received SST for 10 sessions weekly. The results showed that SST had a significant effect on sexual dysfunction scores in the IG compared to the CG (p < 0.001) (14).

In a study that compared the effect of face-to-face and educational booklet teaching methods on anxiety levels and SF among infertile women, 111 were randomly assigned into 3 groups (2 IGs, such as personal training, teaching through booklet distribution, and CGs). The results showed that after the intervention, there were statistically significant differences between IGs and CG in 5 SF domains, such as desire, arousal, lubrication, orgasm, and dyspareunia. This study concluded that both infertility treatments, such as personal training (p < 0.001) or distributing educational booklet (p < 0.0001), were considered effective strategies for infertile women (15).

In the study of Ramadan and co-workers (16), 100 infertile women in the gynecology clinic were recruited in the IG. In this quasi-experimental study, no CG was considered. Results showed that 49.0% of women had poor knowledge before the intervention, but after one month of educational intervention, 73.0% had good knowledge. Also, the mean score of SF among infertile women was statistically significant pre- and post-one month of intervention (p < 0.01). Regarding the effect of educational intervention on attitude regarding the adaptation of infertility, it showed that only 4% had a positive attitude; however, after one month of intervention, 92.0% of infertile women developed a positive attitude.

The results of a study (34), which was performed to determine the effect of sexual education on the SF of women with infertility according to the sexual health model, 108 women with infertility were randomly placed in intervention and CGs. Participants in the IGs received 3 (90 min) sessions of sexual education performed during 1 wk, and the CG received routine care services. The FSFI was completed at the initial stage of the study and one month after the end of the intervention. The results indicated a significant difference in the SF of participants in the IG study before and after the intervention (p < 0.001). In addition, a significant difference was observed between groups regarding the mean score of SF after one month of intervention (p < 0.001).

In a study that evaluates the effect of an infertile couple's skills promotion package on managing sexual relationships, 36 couples with infertility (72 individuals) were assigned randomly to the intervention and CGs. The educational package was presented for couples in the IG simultaneously during 8 sessions. The CG only received infertility treatment during the intervention period. The results showed that the mean scores of SF and sexual adjustment were not significant between the 2 groups before the intervention (p > 0.050), but the mean scores of female SF and sexual adjustment in couples with infertility increased significantly in the IG compared to the CG after the intervention (p < 0.001) (37).

The study, which investigated 577 women between 18-39 yr with infertility and body mass index ≥ 29 kg/m^2^, was randomized into the IG (to a 6-month lifestyle intervention including physical activity, diet, and behavior modification) and the CG received usual care. Results revealed that the IG had more frequency of intercourse (6.6 ± 5.8 vs. 4.9 ± 4.0 times), had higher scores for vaginal lubrication (16.5 ± 3.0 vs. 15.4 ± 3.5), and also had a higher total SF score (96.5 ± 14.2 vs. 91.4 ± 12.8) compared to the CG (p = 0.04). In this study, the mean scores of SF domains, such as those of sexual interest, satisfaction, orgasm, and sex partner, were not statistically different between the 2 groups after the intervention (18).

A study investigated the effectiveness of transactional analysis (TA) therapy on emotion regulation strategies and SF of infertile women. In this study, 30 infertile women referred to infertility clinics were randomly specified to (TA) therapy (n = 15) and CG (n = 15). Participants completed the cognitive emotion regulation questionnaire and FSFI before and after the intervention and 2-month follow-up. The IG received (TA) 2 hr weekly therapy for 8 wk, and the CG received no educational session during the therapeutic intervention. The results of this study showed that after the intervention, TA therapy had a significant effect on the SF of infertile women in the IG compared to the CG (p < 0.05) (17).

#### Psychological counseling or therapy interventions 

A total of 5 included studies investigated the use of the different types of psychological counseling or therapy interventions to improve SF among couples with infertility or infertile women.

In a study, the effect of sexual counseling on the female sexual health of infertile women with sexual dysfunction was assessed; 70 infertile women with primary infertility were assigned to the intervention (n = 35) and CGs (n = 35). The IG received 2 sessions of sexual counseling based on the BETTER model, while routine care was performed for the CG. The results indicated that the counseling sessions improved the total FSFI scores significantly in the experimental group (p = 0.001) compared to the CG (p = 0.557). Also, the results represented that women with 6 or more years of infertility had less improvement in SF and SS (31).

In the quasi-experimental pretest-posttest study assessed the effectiveness of acceptance and commitment therapy on SF of women with infertility, and participants were divided randomly into ACT (n = 15) and CG (n = 15). The FSFI was completed before, after the intervention, and after a 2-month follow-up. The study results indicated that the mean scores of SF in the ACT group significantly increased in the post-test stage (p < 0.01) (32).

The randomized clinical trials assessed the effect of cognitive-behavioural therapy (CBT) on SF of infertile women. In this study, 52 women with infertility were randomly assigned to intervention and CGs (received routine care and education). The participants were given 6 intervention sessions (6-8 individuals in each group, once a week, for 6 consecutive weeks) of sexual counseling using CBT. The SFs of both the intervention and CGs were assessed through FSFI before, after, and one month after the intervention. The results of this study indicated a significant difference in terms of the mean scores of the FSFI between intervention (29.35 ± 2.71) and CG (25.84 ± 2.52) a month after intervention (p < 0.01) (33).

A randomized clinical trial was performed to assess the effectiveness of psychosexual therapy (PST) as an alternative treatment compared with bupropion extended-release for promoting SF among infertile women. In this study, 105 infertile women with sexual dysfunction were randomly allocated to 3 groups: PST, bupropion extended-release, and CGs. The women in the PST group participated in weekly 2-hr sessions for 8 consecutive weeks. Also, the BUP ER group received 150 mg/day of Bupropion ER for 8 wk, and the CG only received routine care. In this study, the mean scores of FSFI and its subscales increased significantly in PST (p = 0.0001) and BUP ER (p = 0.0001) groups, except in the subscale of sexual pain) compared to the CG. Also, results comparing the 2 intervention methods showed that PST had a better effect on improving SF than BUP ER (p = 0.0001) (35).

The effect of sexual counseling on the SF of women with infertility was investigated. This study randomly assigned participants to intervention (2 sessions for 60-90 min of sexual counseling in a private room) and CGs (routine care). Participants at baseline completed the FSFI 2 months after the intervention. The results showed that all subscales of FSFI except the pain domain were significantly increased in the IG after 2 months (p < 0.001) compared to the CG (36).

### The interventions regarding SS among infertile individuals 

The interventions of the included studies were systematically classified into 2 main categories, which were reported below:

#### Educational interventions 

A total of 2 included studies investigated the use of different educational methods to improve SS among couples with infertility or infertile women. In the RCTs study (23), the effect of a marital relationship enrichment program (MREP) on marital satisfaction and SS of 50 couples with infertility had been evaluated. The intervention, including g MERP, was presented to the IG for 90 min during 7 sessions. The related questionnaires were completed before and after the training and also 8 wk later for the follow-up stage. The posttest and follow-up mean scores showed that MERP significantly increased SS among the IG compared to the CG (p < 0.001). The study by Shahbazi and colleagues (26) investigated the effect of an educational program on the quality of sexual relationships among 80 infertile women. The intervention was taught to participants based on the BASNEF model during four 60-90 min sessions. The results indicated that satisfaction with the quality of sexual relationships had significantly increased in the IG group compared to the CG after the intervention (p < 0.05).

#### Psychological counseling or therapy interventions 

In 8 studies, different psychological counseling or therapy interventions were performed to assess SS among couples with infertility or infertile women. In a controlled clinical trial that evaluated the effect of counseling on SS among infertile women, the results revealed that despite there being no differences regarding SS among the 2 groups (p = 0.401) before the intervention, a significant difference was seen 3 months after the intervention (p = 0.019) (19). Also, another study investigated the effect of 2 counseling on the SS of infertile women; mean differences comparison between the 2 groups showed the effectiveness of collaborative infertility counseling among women with infertility (p = 0.027) (24).

In 2 studies, the effect of psychological interventions on marital satisfaction and SS among couples with infertility were assessed, respectively. In the first study, performed on 70 couples with infertility in each group, psychological treatment such as CBT and supportive psychotherapy were performed for 6 months on the IG. A significant decrease in marital satisfaction over time in the CG and a significant increase in the IG (p < 0.001) were observed (20). The second study investigated the effects of a psychological intervention program on 50 women with infertility. The SS as an outcome measure was measured at baseline and 4 wk after the end of the intervention. The results indicated that psychological intervention increased SS significantly in the experiment group (p = 0.003) (27).

2 Iranian studies evaluated the effectiveness of integrative positive-cognitive and integrative behavioural couple therapy (IBCT) on SS among infertile women. In the first study, the intervention was performed for the experimental group for 10 sessions of 90 min using the positive-cognitive behavioral therapy approach, and the CG received only the routine care. The results showed the effectiveness of this method in improving the SS among infertile women who participated in the IG compared to the CG (p < 0.01) (25). In the second study, integrative behavioral couple therapy was performed for participants in the IG for 120 min for 12 sessions. This study was a single case experimental study in which the second couple entered into the study in the second session of the first couple and the third couple enrolled in the treatment plan in the third session of the first couple, and the second session of the second couple. The results showed that the IBCT increased infertility SS (55.01%) in couples with infertility (28). A quasi-experimental study in Iran investigated the effect of stress management based on group CBT on marital satisfaction among infertile women. The intervention was presented to 20 infertile women in the experimental group during a 10 (2 hr) sessions during the 2-month intervention and 3-month follow-up. The results showed that the mean marital satisfaction score was significantly higher in the experimental group compared to the CG in both posttest (p = 0.001) and follow-up (p = 0.001) (22). A quasi-experimental study investigated the effect of emotionally focused couples therapy (EFT-C) on the SS of couples with infertility and marital conflicts. The results indicated that EFT-C significantly increased the SS among the IG compared to the CG (p = 0.027) (21).

### Quality appraisal of included studies 

In table III, the strengths and weaknesses of each study were shown. Only 2 studies were low quality (33, 38), whereas others had satisfactory results (moderate and high quality).

## 4. Discussion

This systematic review assessed the psychological or educational intervention studies emphasizing the SF and SS among infertile women or couples. The literature review showed that different interventions had been conducted on SF and SS among infertile individuals, most of which were carried out in the Iranian samples. All included studies in this current systematic review showed that their interventional sessions had a significant therapeutic effect on women's sexual issues. Regarding the SF, most interventions were educational, such as CST, workshop psychoeducational sessions, SST, personal training and booklet distribution strategies, educational TA therapy, and lifestyle intervention. Regarding SS, most interventions were psychological, such as counseling, stress management based on group CBT, MREP, and collaborative infertility counseling.

In general, having a desirable sexual relationship can increase the probability of fertility and lead to appropriate psychological well-being. Studies have shown that sexual problems and disorders should be considered an essential part of the evaluation among couples with infertility (1, 10). Despite the high prevalence of sexual complaints among infertile women referred to gynecology settings, the women are less interested in discussing their problems, which the physicians usually miss (38). In this regard, studies have shown that SFs and depressive symptoms frequently require less attention during infertility treatment because healthcare providers generally emphasize conception and have limited knowledge and training regarding the treatment of sexual health (38, 39).

Diagnosis of infertility, infertility treatment, and the failure of infertility treatment may lead to a high prevalence of sexual dysfunction among infertile women (10, 38), so several studies all over the world with similar and different cultural statuses showed that sexual dysfunction among infertile women was calculated 26.0-76.5% that is significantly higher than fertile women (10, 40). The cultural and social pressure and stigma of infertility were the main issues that negatively affected the psychological status and sexual life of infertile women (41).

Several studies regarding sexual problems in infertile women have also shown that sexual desire, arousal, orgasm, dyspareunia, and vaginismus were the most common sexual problems among couples with infertility (42, 43). Also, another study on infertile women referred to 12 infertility clinics in 5 cities in Iran showed that infertile women had significantly lower scores in different dimensions of FSFI compared to fertile women (44).

Couples with infertility worried about how infertility and associated treatments affect their SF and SS, as well as higher sexual dysfunction rates among infertile women than fertile women. This showed the effectiveness of psychological interventions to cope with or alleviate stress and sexual problems associated with infertility along with the main assisted reproductive treatments (1). A study in the United States showed that infertility can negatively affect SF, quality of life, and psychological well-being (45). In this systematic review, 2 important sexual domains, SF and SS, were reviewed.

According to the mentioned sexual complaints among couples with infertility, various interventional studies were recently conducted to improve their SF. Among included studies, most of them were psychological interventions such as CBT (33), integrative behavioral couple therapy (28), emotionally focused couple therapy (21), sexual counseling (19, 31, 36), MREP (23), and ACT (32) that showed the effective therapeutic outcomes. SF is an affected aspect of infertile women that has been investigated in the studies (46). Various factors such as the couple's psychological status, sex hormone status, age of participants, duration of the marriage and adverse effects of medications, economic problems such as the costs of diagnosis and treatment of infertility are considered as possible causes of sexual dysfunction among couples with infertility (42, 46). In general, SF was positively affected by SS, so SS led to positive emotions, belief regarding sexual communication, and also a positive attitude regarding SF (4).

SS is considerably affected by infertility outcomes such as feelings of depression, failure in conceiving, and decreased self-esteem (10) and chronic diseases and medication (10). Studies about the level of SS among couples with infertility revealed contradictory results. All of the included studies regarding SS and activity in this systematic review showed that couples with infertility had a reduced level of SS following the infertility diagnosis. The administered interventions increased the SS among couples with infertility compared to the CG (22, 26, 27). Psychological pressure on both men and women to have a child negatively affects their sexual intimacy and satisfaction (39, 47). In contrast, a study declared that SS and SF did not reduce considerably in infertile women compared to the normal population (48).

### Strength and limitations

This study can be a good resource for psychologists to review and assess the current RCTs or quasi-experimental studies on SF and SS and select the best intervention studies for managing their patients.

The major strength of this study was a systematic review of the published interventional research on SF and SS among infertile individuals using the PRISMA guidelines.

## 5. Conclusion

Based on this systematic review, different interventions had been used for increasing the SF and SS among couples with infertility, and all of the included studies had shown effectiveness in this regard. Couples with infertility are exposed to various individual, interpersonal, and psychosocial stressors that may negatively affect their SF and SS. The results of this systematic review showed that regarding the SF, most of the studies were educational and regarding the SS, most were psychological interventional studies. Also, the results indicated that most published studies were conducted in the Iranian population. There was a considerably low published literature in other countries, so conducting standardized and high-quality RCTs focusing on SF and SS is recommended.

##  Conflict of Interest

The authors declare that there is no conflict of interest.
